# isomiRs: Increasing Evidences of isomiRs Complexity in Plant Stress Functional Biology

**DOI:** 10.3389/fpls.2015.00949

**Published:** 2015-11-10

**Authors:** Gaurav Sablok, Ashish K. Srivastva, Penna Suprasanna, Vesselin Baev, Peter J. Ralph

**Affiliations:** ^1^Plant Functional Biology and Climate Change Cluster (C3), University of Technology SydneySydney, NSW, Australia; ^2^Nuclear Agriculture and Biotechnology Division, Bhabha Atomic Research CentreMumbai, India; ^3^Department of Plant Physiology and Molecular Biology, University of PlovdivPlovdiv, Bulgaria

**Keywords:** biotic and abiotic stress, development, miRNAs, plants, isomiRs, smallRNA-seq

## miRNAs and isomiRs: rise of complex miRNA isoforms

How and by which mechanism plants control post-transcriptional regulation? Reinhart et al. (2002) pinpointed the role of non-coding, small endogenous regulatory microRNAs (miRNAs) as the regulatory switch that controls the post-transcriptional regulation. Since then, several families of regulatory miRNAs including artificial miRNAs (Sablok et al., 2011) have been discovered and have shown to play a key role as system wide regulators, which are activated in response to several biotic and abiotic stress adaptations (Zhang, 2015), as well as regulators of plant development (Reinhart et al., 2002; Sun, 2012). Several reports on the identification and characterization of miRNAs have been published so far (for a review, see Budak et al., 2015), which established miRNAs as major post-transcriptional regulatory factors in plants system biology. Endogenous miRNAs perform regulatory role by binding specifically to the 3′-UTR of mRNAs, which in turns triggers the degradation of the targeted transcript, thereby leading to the shutdown of protein translation. Endogenous miRNAs originate from the stem of a single-stranded stem-loop precursor as a miRNA/miRNA^*^ duplex with an approximately 2-nt 3′-end overhang. However, not all small RNAs expressed from a stem-loop are necessarily either the precise miRNA or precise miRNA^*^.

The advent of the high throughput small RNA sequencing approaches and concurrent development in the identification of miRNAs using advanced algorithmic-based computational approaches has led to the discovery of a new class of regulatory RNAs called isomiRs, which are canonical variants of miRNAs (Morin et al., 2008b). The biogenesis of these canonical miRNA sequence variants in plants might be due to the imprecise cleavage activity by the Rnase III enzyme or due to the post-transcriptional RNA editing events (Hackenberg et al., 2013) or by nucleotidyl transferases (Wyman et al., 2011). Morin et al. (2008a) hypothesized the origin of isomiRs as cleavage site variability in the pre-miRNA hairpin, which is cleaved by either DICER1 or DROSHA (Morin et al., 2008b).

Taking into account the length and nucleotide variation along with the non-templated additions (NTAs) such as adenylation and uridylation at the 3′-end with non-random functionality (Wyman et al., 2011), isomiRs can be classified into 5′ isomiR, 3′ isomiR, and polymorphic isomiRs (Neilsen et al., 2012; Jeong et al., 2013). Global analysis of the isomiRs and canonical miRNAs has shown uridine to be the preferential nucleotide at 5′- and 3′-ends. This has led to the conclusion that isomiRs commonly feature “U–C” at the 3′-end of the isomiRs as opposed to the addition of C at the 3′-end of the plant miRNAs (Zhang et al., 2013). However, isomiRs displayed a frequent truncation of the cytodine from both the ends, presenting a new complex cytodine balance in isomiRs (Xie et al., 2015), which suggests that uridylation plays a role in avoiding degradation. The primary sequences of identified isomiRs so far differ from the indexed high content miRBase miRNAs in their 5′-end (changing the “seed” region and suggesting a different target molecule) or in their 3′-end or both. The question that why this variant occurs is still intriguing and challenging, and needs to be addressed. However, recent reports have started suggesting the target cleavage efficiency of isomiRs, thus establishing them as another class of regulatory functional RNAs.

## isomiRs identification and inter-play in plant stress functional genomics

On the basis of accumulated knowledge about isomiRs, as well as their abundance and involvement in target cleavage (Ahmed et al., 2014), several algorithms either as web-based or standalone tool have been developed, using read mapping approaches to delineate the repertoire of isomiRs abundance and expression (Table 1). Previously, we developed the first web-based tool, isomiRex (bioinfo1.uni-plovdiv.bg/isomiRex/), which provides the high-throughput classification and differential expression of isomiRs and supports a broad range of organisms including plants (Sablok et al., 2013). Following isomiRex, several tools have been developed such as IsomiRage (Muller et al., 2014) and isomiRID (de Oliveira et al., 2013), which can distinguish isomiRs using template-based and non-template-based predictions. Since isomiRs can be a result of the adenylation or uridylation events, IsomiRage (Muller et al., 2014) implements algorithmic identification and classification of functionally relevant isomiRs based on their adenylation, uridylation, and other respective biological events (Muller et al., 2014).

**Table 1:** **Recently developed classification tools for identifying isomiRs**.

**Tool**	**Address**	**Type**	**References**
SeqCluster	https://github.com/lpantano/seqbuster	Standalone	Pantano et al., 2011
miRSeqNovel	http://sourceforge.net/projects/mirseq/files	Standalone	Qian et al., 2012
isomiRID	http://www.ufrgs.br/RNAi/isomiRID/	Standalone	de Oliveira et al., 2013
isomiRex	bioinfo1.uni-plovdiv.bg/isomiRex/	Web-based	Sablok et al., 2013
miRspring	http://mirspring.victorchang.edu.au	Standalone	Humphreys and Suter, 2013
IsomiRage	http://cru.genomics.iit.it/Isomirage/	Standalone	Muller et al., 2014
sRNAtoolbox	http://bioinfo5.ugr.es/srnatoolbox	Standalone	Rueda et al., 2015

Whether isomiRs are functional or just variants has been widely discussed and debated since the first reported evidence of isomiRs in *Oryza sativa* (Morin et al., 2008b). Although their functional role and capabilities are still unknown, isomiRs have the potential to extend the canonical miRNA regulatory network. This adds credence to the hypothesis that “mature miRNAs” cannot be related to only a single individual sequence and that a single precursor may cleave more than one functional product. Profiling of tissue-specific small RNAs in Peach (*Prunus Persica L*.) revealed an abundance of tissue-specific isomiRs (392 isomiRs—miRNA and miRNA^*^-related—corresponding to 26 putative miRNA coding loci), which supports the hypothesis that the origin of isomiRs is not just a random functional event in plant post-transcriptional machinery (Colaiacovo et al., 2012). Realizing the potential of isomiRs and their ability to cleave targets (Ahmed et al., 2014), several studies started exploring their functional role in plants. In *Phaseolus vulgaris*, as many as 57 functional isomiRs spanning across 25 families have been identified in nodule development and phasiRNAs generation (Formey et al., 2015).

Alongside the discovery of isomiRs and their ability to cleave targets, studies pertaining to the relative differential expression of isomiRs have also been conducted. Jeong et al. (2013) reported strong expression of isomiRs with 5′ variations in *A. thaliana* using Parallel Analysis of RNA Ends (PARE-Seq), co-immunoprecipitation, and *ARGONAUTE* (AGO) loading data (Jeong et al., 2013). Using a reversed framework approach and degradome analysis, Shao et al. (2015) revealed the higher expression of the isomiRs as compared to their canonical miRNAs in *Oryza sativa* (Shao et al., 2015). Interestingly, they showed high abundance of iso-osa-miR528-5p in AGO1 complexes as compared to the osa-miR528-5p (1315 rpm vs. 165.72 rpm) (Shao et al., 2015). Ehya et al. (2013) observed differential expression of several isomiRs of canonical miRNAs involved in auxin signaling, such as miR160 (for miR160^*^), miR166, and miR167 suggesting miRNA-mediated auxin signaling, which might regulate the response of the Mexican Lime tree to Phytoplasma (Ehya et al., 2013). Taking these studies into account, it can be concluded that isomiRs are not random events and might play a key role in increasing the miRNAome complexity in plants.

In addition to the developmental and tissue-specific functions (Colaiacovo et al., 2012), isomiRs play an important role in modulating and regulating the miRNAome in biotic and abiotic stress conditions. However, as compared with the widely demonstrated regulatory roles of the differentially expressed, conserved and novel miRNAs in biotic and abiotic stress conditions in plants (Zhang, 2015), limited reports have shown the differential expression of the isomiRs (Baev et al., 2014). Nonetheless, it is worthwhile to mention that recent reports in stress conditions have presented the evidences of isomiRs expression along with the expression of the canonical miRNAs in model plants. The information gleaned from the recent phosphorus (P) deficiency stress studies in *Hordeum vulgare* (Barley) have highlighted the up-regulated isomiRs of miR399 and miR827 family under P deficiency significantly (Hackenberg et al., 2013). Understanding the post-transcriptional regulation of the signaling pathways during temperature stress response is critical in dealing global climate change. Baev et al. (2014) recently demonstrated the differential regulation of the miR160c isomiRs in high- and low-temperature conditions in *Arabidopsis thaliana* with the identified isomiRs showing substantially higher expression as compared to the canonical miRNAs. Such regulated isomiRs under P deficiency and high- and low-temperature stress suggest that isomiRs play a role in regulating the miRNAome in stress-induced biological gene regulation.

In addition to the relative recent increase in the knowledge gain about isomiRs, their biogenesis and expression, significant efforts have been made to understand the role of the functional target cleavage capacity of the isomiRs. Since reported variation in isomiRs sequences occurs at the 3′- or 5′-ends, they could potentially bind to a different repertoire of targets relative to their mature reference counterparts. The higher expression of the isomiRs as compared to the canonical mature miRNA may affect the target cleavage efficiency of that particular miRNA. Using PARE-Seq, Jeong et al. (2013) demonstrated the differential target cleavage capacity of miR161.1 isomiRs. In model plant *A. thaliana*, differential binding capacities of the isomiRs as compared to the canonical miRNAs, as well as high efficiency of the isomiRs in target cleavage as compared to the canonical miRNAs, have been recently demonstrated (Ahmed et al., 2014). In addition, higher target prediction efficiency using coupled combinations of miRNAs and isomiRs has been observed in *A. thaliana* (Ahmed et al., 2014). Although less evidence have been shown toward the functional gain or loss of the isomiRs in plants, they have been confirmed experimentally in other models systems including humans, thus representing the role of isomiRs as evolutionary and functionally important variants (Tan et al., 2014; Cammaerts et al., 2015). In conclusion, isomiRs act as canonical partners to miRNAs in regulating developmental and stress-associated post-transcriptional responses in stress. Realizing the emerging occurrences and the read-based support for the detection of the isomiRs, it can be presumed that post-transcriptionally isomiRs and canonical miRNAs act synergistically to regulate the developmental and signaling pathways in plants.

## Authors contributions

GS and AS conceived the idea, GS drafted the opinion article, PS, VB and PR provided revisions to the article.

### Conflict of interest statement

The authors declare that the research was conducted in the absence of any commercial or financial relationships that could be construed as a potential conflict of interest.
